# Supplementary material for: A UMLS-based spell checker for natural language processing in vaccine safety
Source: BMC Med Inform Decis Mak. 2007 Feb 12;7:3. doi: 10.1186/1472-6947-7-3 (PMC1805499; doi:10.1186/1472-6947-7-3)
Supplement: Additional file 13 — RAP application directory. Sets up directory for RAP (RDF application for PHP) source code [file 1472-6947-7-3-S13.gz › rap/doc/phpdoc/__filesource/fsource_model__modelLiteral.php.html]

xml version="1.0" encoding="iso-8859-1"?


File Source for Literal.php


# Source for file Literal.php

Documentation is available at Literal.php

```
1. <?php

3. // ----------------------------------------------------------------------------------
4. // Class: Literal
5. // ----------------------------------------------------------------------------------


9. /**
10. * An RDF literal.
11. * The literal supports the xml:lang and rdf:datatype property.
12. * For XML datatypes see: http://www.w3.org/TR/xmlschema-2/
13. *
14. * <BR><BR>History:<UL>
15. * <LI>11-08-2003                 : Method setLanguage() added</LI>
16. * <LI>12-04-2002                 : Datatype support added ($dtype, getDatatype,
17. *                                    toString, setDatatype, equals)</LI>
18. * <LI>09-10-2002                : First version of this class.</LI>
19. *
20. * @version  V0.9.1
21. * @author Chris Bizer <chris@bizer.de>
22. * @author Daniel Westphal <dawe@gmx.de>
23. *
24. * @package model
25. * @access    public
26. *
27. */
28. class Literal extends Node {

30. /**
31. * Label of the literal
32. * @var        string
33. * @access    private
34. */
35. var $label;
36. /**
37. * Language of the literal
38. * @var        string
39. * @access    private
40. */
41. var $lang;

43. /**
44. * Datatype of the literal
45. * @var        string
46. * @access    private
47. */
48. var $dtype;


51. /**
52. * Constructor
53. *
54. * @param    string    $str            label of the literal
55. * @param     string $language        optional language identifier
56. *
57. */
58. function Literal($str, $language = NULL) {
59. $this->dtype = NULL;
60. $this->label = $str;

62. if ($language != NULL) {
63. $this->lang = $language;
64. } else {
65. $this->lang = NULL;
66. }


69. }

71. /**
72. * Returns the string value of the literal.
73. *
74. * @access    public
75. * @return    string value of the literal
76. */
77. function getLabel() {

79. return $this->label;
80. }

82. /**
83. * Returns the language of the literal.
84. *
85. * @access    public
86. * @return    string language of the literal
87. */
88. function getLanguage() {

90. return $this->lang;
91. }

93. /**
94. * Sets the language of the literal.
95. *
96. * @access    public
97. * @param        string $lang
98. */
99. function setLanguage($lang) {

101. $this->lang = $lang;
102. }

104. /**
105. * Returns the datatype of the literal.
106. *
107. * @access    public
108. * @return    string datatype of the literal
109. */
110. function getDatatype() {

112. return $this->dtype;
113. }

115. /**
116. * Sets the datatype of the literal.
117. * Instead of datatype URI, you can also use an datatype shortcuts like STRING or INTEGER.
118. * The array $short_datatype with the possible shortcuts is definded in ../constants.php
119. *
120. * @access    public
121. * @param        string URI of XML datatype or datatype shortcut
122. *
123. */
124. function setDatatype($datatype) {
125. GLOBAL $short_datatype;
126. if  (stristr($datatype,DATATYPE_SHORTCUT_PREFIX))  {
127. $this->dtype = $short_datatype[substr($datatype,strlen(DATATYPE_SHORTCUT_PREFIX)) ];}
128. else
129. $this->dtype = $datatype;
130. }

132. /**
133. * Checks if ihe literal equals another literal.
134. * Two literals are equal, if they have the same label and they
135. * have the same language/datatype or both have no language/datatype property set.
136. *
137. * @access    public
138. * @param        object    literal $that
139. * @return    boolean
140. */
141. function equals ($that) {

143. if (($that == NULL) or !(is_a($that, 'Literal'))) {
144. return false;
145. }

147. if ( ($this->label == $that->getLabel()) && ( ( ($this->lang == $that->getLanguage()) ||
148. ($this->lang == NULL && $that->getLanguage() == NULL) )  &&

150. (
151. ($this->dtype == $that->getDatatype() ||
152. ($this->dtype == NULL && $that->getDatatype() == NULL)) ) ) )
153. {
154. return true;
155. }

157. return false;
158. }

160. /**
161. * Dumps literal.
162. *
163. * @access    public
164. * @return    string
165. */
166. function toString() {
167. $dump = 'Literal("' . $this->label .'"';
168. if ($this->lang != NULL)
169. $dump .= ', lang="' . $this->lang .'"';
170. if ($this->dtype != NULL)
171. $dump .= ', datatype="' . $this->dtype .'"';
172. $dump .= ')';
173. return $dump;
174. }


177. } // end: Literal

179. ?>
```

Documentation generated on Thu, 7 Jul 2005 13:41:52 +0200 by phpDocumentor 1.3.0RC3
